# 2270. Dealing with an old foe –Assessment of Knowledge, Attitude, and Practices: A survey on management of *Staphylococcus aureus* bacteraemia among Indian Physicians

**DOI:** 10.1093/ofid/ofad500.1892

**Published:** 2023-11-27

**Authors:** Akshatha Ravindra, Deepak Kumar, Durga Shankar Meena, Santhanam Naguthevar, Gopal Krishana Bohra

**Affiliations:** AIIMS Jodhpur, Jodhpur, Rajasthan, India; All India Institute of Medical Sciences, Jodhpur (India), Jodhpur, Rajasthan, India; AIIMS, Jodhpur, Rajasthan, India; AIIMS JODHPUR, Jodhpur, Rajasthan, India; AIIMS Jodhpur, Jodhpur, Rajasthan, India

## Abstract

**Background:**

*Staphylococcus aureus* bacteraemia (SAB) is associated with high morbidity, mortality, and healthcare costs. Effective management of SAB significantly improves outcome. There is significant gap in the guidelines and clinical practice. This survey was conducted with aim to assess opinions and practice patterns in management of SAB among Indian clinicians.

**Methods:**

We conducted a cross-sectional respondent-driven survey, using a 45-item, anonymous, voluntary, multiple-choice google form questionnaire among clinicians in India, after obtaining ethical approval from the institute. We distributed survey via social media, WhatsApp, and E-mail. Survey responses were analysed using SPSS, Version 25.0.

**Results:**

The total of 125 practicing clinicians responded to this survey. Questions where systematically divided into treatment of choice for MSSA, MRSA, additional investigations, persistent bacteraemia, and adjunctive anti-toxin therapy. Around 57% of the respondents have 1-10 episodes of SAB every month. For suspected SAB, most respondents (74%) preferred vancomycin as empiric therapy. If MSSA was identified, 66.7% initiated on Anti staphylococcal penicillin, while in MRSA vancomycin was selected by majority (80%) (Figure 1 & 2). Most clinicians (74%) perceived 1st generation cephalosporins non-inferior to ASPs. Infectious diseases referral for SAB was undertaken in only in about 56% of the cases, with 25% respondents citing non-availability of ID specialist in the institute. Repeat blood cultures and echocardiography was performed by most respondents. Lack of consensus was apparent in diagnosis and management of persistent bacteraemia and oral switch of therapy (Figure 1 & 2).Most respondents managed SAB with at least 14 days of parenteral antibiotics in several scenarios. But, in patients with complicated SAB there was significant practice variation (Figure 3).

Figure - 1

**Figure:**
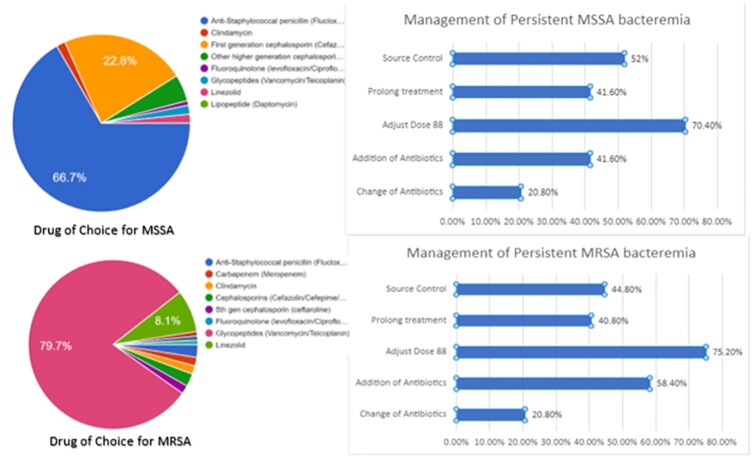

Drug of choice in MSSA, MRSA and management of persistent bacteraemia according to the respondents

Figure 2

**Figure:**
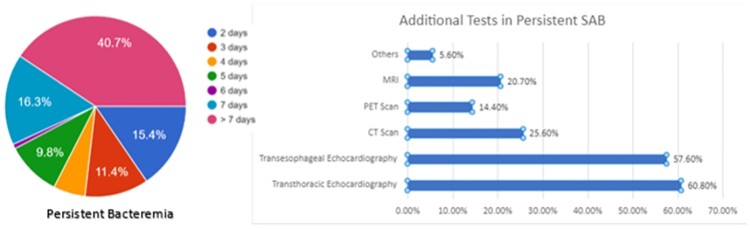

Persistent bacteraemia definition according to the respondents along with the additional tests performed

**Conclusion:**

There were some areas of consensus, but this survey highlights considerable practice variation among clinicians. Although the value of ID consultation in SAB management has been established by multiple studies, this survey demonstrates that there remains ample opportunity to further define best practices and optimize management of this complex disease.

**Disclosures:**

**All Authors**: No reported disclosures

